# Clathrin in *Chara australis*: Molecular Analysis and Involvement in Charasome Degradation and Constitutive Endocytosis

**DOI:** 10.3389/fpls.2017.00020

**Published:** 2017-01-26

**Authors:** Marion C. Hoepflinger, Margit Hoeftberger, Aniela Sommer, Christina Hametner, Ilse Foissner

**Affiliations:** Department of Cell Biology and Physiology, Faculty of Natural Sciences, University of Salzburg, Salzburg, Austria

**Keywords:** *Chara*, charasome, clathrin, plasma membrane, endocytosis, ikarugamycin

## Abstract

Charasomes are convoluted plasma membrane domains in characean green algae. They are known to form in response to light via secretion of *trans*-Golgi network (TGN) vesicles and local inhibition of endocytosis. Charasomes are involved in the acidification of their aqueous environment, thereby facilitating photosynthesis-dependent carbon uptake. Charasome formation is reversible to allow cells to adapt to different light conditions. Here, we show that darkness-induced degradation of charasomes involves the formation of coated pits and coated vesicles. The darkness-induced degradation of charasomes can be inhibited by 1–2 μM ikarugamycin (IKA), which is considered to be a specific inhibitor of clathrin-dependent endocytosis. At a much higher concentration (100 μM), IKA also significantly reduces the internalization of styryl dyes, indicating uptake via clathrin-coated vesicles (CV). We are the first to present evidence, based on fine structure investigation, that IKA does not interfere with the formation of clathrin coat, but inhibits the detachment and/or further processing of coated vesicles. Both charasome degradation and constitutive endocytosis are also significantly inhibited by sterol complexing agents (methyl-ß-cyclodextrin and filipin). The absence of an additive effect, when applied together with IKA, suggests that charasome degradation and constitutive endocytosis (measured via styryl dye uptake) is not inhibited due to membrane retrieval via lipid rafts, but due to clathrin coat formation requirement of a specific set of sterols. Analysis of *Chara australis* clathrin proteins revealed two heavy chains and several light chains with sequence peculiarities, suggesting functional and/or species specific differences. The data obtained indicate that clathrin plays a central role not only in constitutive endocytosis but also in the degradation of charasomes, thereby representing a valuable system for studying targeted exo- and endocytosis.

## Introduction

Exo- and endocytosis are vital processes required for growth, plasma membrane recycling and repair, uptake of nutrients, and signaling. Both pathways can be classified according to their cargo, as well as the proteins and lipids involved in vesicle production and trafficking. Additionally, the induced and constitutive endo- and exocytosis can be distinguished, depending on whether an external signal is required for the induction of these pathways or not. Numerous studies have shown that clathrin-dependent endocytosis is the main pathway for plasma membrane recycling in plant cells (e.g., Konopka et al., 2008; Kitakura et al., 2011; Wang et al., 2013, 2016).

Clathrin-dependent endocytosis is initiated at the plasma membrane with the recruitment of coat machinery and cargo proteins into clathrin-coated membrane invaginations the so called clathrin coated pits (CPs). Several models of CP formation are described (for review see Chen et al., 2011; Fan et al., 2015 and references therein). On the one hand, the highly conserved adaptor protein complex 2 (AP2) is described as a central player in the initiation of CP nucleation. AP2 and accessory proteins recruit clathrin subunits directly from the cytoplasm to the nucleation site. Clathrin subunits form hexameric, three legged triskelia by self-polymerization. The triskelia comprise three clathrin heavy chains (CHCs) and three clathrin light chains (CLCs). On the other hand a well-defined CP initiation site could pre-exist on the plasma membrane before AP2 recruitment. The latter is well-described in animals and yeast, but has not yet been identified in plants. However, after CP nucleation the pits mature via cargo-selection by a precise sorting machinery, where cargo-selective clathrin adaptors specifically bind to sorting motifs of cargo proteins. Besides AP2 complex several other conserved orthologs of adaptor proteins have been identified in plants: The epsin N-terminal homology domain, the AP180 N-terminal homology domain containing proteins and an epsin-like clathrin adaptor1 (AtECA1), which may act as adaptor protein in coat assembly (Fan et al., 2015 and references therein). Specific cargo proteins for AtECA1 during clathrin-mediated endocytosis (CME) have not been identified in plants so far. After gathering the trans-membrane cargo, further clathrin coat and accessory proteins are assembled and finally the clathrin-coated vesicle (CV) is pinched off. The GTPases dynamin and dynamin-related proteins (DRPs) mediate membrane tubulation and scission (e.g., Konopka et al., 2008; Backues et al., 2010; Fujimoto et al., 2010). Cytosolic CVs are quickly uncoated, and fuse with early endosomes where the cargo is further sorted for either recycling (back to plasma membrane) or degradation (transport to the vacuole). Unlike in animal cells, plant CVs are not only formed on the plasma membrane, but also in the trans-Golgi network (TGN), where they are involved in various post-Golgi trafficking processes (Robinson and Pimpl, 2014).

We have previously documented that internodal cells of Characean green algae are a valuable model for the study of wound healing, and therefore also for induced exo- and endocytosis (Foissner et al., 2015). Depending on the severity of damage, wounds are healed in the presence or absence of endocytosis (membrane recycling) thereby determining type and properties of the deposited material. Here, we introduce a new model for studying vesicular trafficking in *Chara* internodal cells: the reversible formation of charasomes.

Charasomes are convoluted plasma membrane domains of multicellular Characean green algae. The first description of charasomes was published independently by Barton and Crawley in 1965 (Barton, 1965a,b; Crawley, 1965). Several years later, detailed electron microscopy studies about their formation and morphology followed (Franceschi and Lucas, 1980, 1981; Lucas and Franceschi, 1981; Lucas et al., 1986; McLean and Juniper, 1986; Bisson et al., 1991; Chau et al., 1994). The use of fluorescent plasma membrane dyes greatly facilitated analysis of charasome abundance and distribution and confirmed that both are depended on the age of branchlet internodal cells, as well as on growth conditions and especially on the light intensity to which they are exposed (Schmoelzer et al., 2011). A more recent study showed that formation of charasomes is not a unique property of internodal cells but that nodal cells and rhizoids are also able to develop these domains (Foissner et al., 2015).

Under normal growth conditions charasomes are not evenly distributed along the cell surface. Extended regions with large, numerous charasomes alternate with smaller areas containing fewer, small charasomes (Franceschi and Lucas, 1980; Bisson et al., 1991; Schmoelzer et al., 2011). Furthermore, the distribution of charasomes correlate with the pattern of acid and alkaline regions along the surface of branchlet internodal cells. This so called banding pattern can be visualized by phenol red (Franceschi and Lucas, 1980; Price et al., 1985; Schmoelzer et al., 2011). It is assumed that this correlation between pH banding pattern and charasome area fraction is due to the high number of H^+^-ATPases accommodated in charasomes, which provide increased area of plasma membrane (Keifer et al., 1982; Price and Whitecross, 1983; Schmoelzer et al., 2011). These H^+^-ATPases acidify the extracellular environment in order to allow the poorly membrane permeable hydrogen carbonate (${\text{HCO}}_{3}^{-}$, bicarbonate) to be reduced to CO_2_. CO_2_ easily diffuses into the cytoplasm where it can be used for photosynthesis (Price and Badger, 1985; Plieth et al., 1994; Bulychev et al., 2001; Ray et al., 2003).

Formation and morphology of charasomes have been described in detail (Franceschi and Lucas, 1980, 1981; Lucas and Franceschi, 1981; Lucas et al., 1986; McLean and Juniper, 1986; Bisson et al., 1991; Chau et al., 1994). According to current knowledge, charasomes are formed by exocytosis of TGN-derived material (Pesacreta and Lucas, 1984; Foissner et al., 2016). During this growth process, the cytoplasm-facing membrane of charasomes bears numerous CPs. These CPs are not released as CVs and thus are probably providing the membrane curvature required for plasma membrane fusion into the complex meshwork of anastomosing charasome tubules (Franceschi and Lucas, 1980; Lucas and Franceschi, 1981; see also Franceschi and Lucas, 1982; Pesacreta and Lucas, 1984; Chau et al., 1994). Fully grown, mature charasomes are largely devoid of coated regions. Therefore, plasma membrane recycling via CPs and CVs seems to be restricted to the smooth plasma membrane regions (non-convoluted) between charasomes (Lucas and Franceschi, 1981; Schmoelzer et al., 2011; Sommer et al., 2015).

During the course of this study, we investigated the degradation of charasomes and compared it with constitutive endocytosis at smooth plasma membrane regions. We show that dark treatment causes increased appearance of a clathrin coat at charasome membranes as well as recycling via CVs. Inhibitor treatments, which point to a central role of clathrin in the formation and degradation of these peculiar organelles prompted us to analyze the sequence of *Chara australis* clathrins. This work revealed several versions of the CLC perhaps indicating functional differences.

## Materials and methods

### Algal material, culture conditions, and inhibitor treatments

Thalli of *C. australis* were grown in a substrate of soil, peat, and sand in 10–50 L aquaria filled with distilled water at a temperature of about 20°C. Fluorescent lamps provided a 14/10 h light/dark cycle. Light intensity was low (about 5 μE.m^−2^.s^−1^) in order to prevent calcification and growth of epiphytes. After several weeks of growth, branchlet internodal cells from the 3^rd^ to the 5^th^ whorl were isolated with a small pair of scissors and left in artificial fresh water (10^−4^ M NaCl, 10^−4^ M KCl, 10^−3^ M CaCl_2_) until use.

Inhibitor stock solutions used during the course of this study were ikarugamycin (IKA; Santa Cruz Biotechnology, California, USA; 10 mM in DMSO), filipin III complex (Sigma-Aldrich, St. Louis, USA; 30 mM in DMSO) and methyl-ß-cyclodextrin (MßCD; Sigma; 20 mM in distilled water). Working solutions were diluted with artificial fresh water. Solvent concentration was the same for all working solutions of one substance and this concentration was also used in control solutions. The pH of artificial fresh water was not significantly altered by addition of inhibitors.

### *In vivo* staining, confocal laser imaging, and statistical analysis

For *in vivo* staining of charasomes and endosomes, internodal cells were pulse labeled for 5 min with fluorescent FM1-43FX (N-(3-triethylammoniumpropyl)-4-(4-(dibutylamino)styryl)pyridinium dibromide; Invitrogen, Carlsbad, USA) and AM1-44 (Biotium, Hayward, USA) or with fluorescent FM4-64 (N-(3-triethylammoniumpropyl)-4-(6-(4-(diethylamino) phenyl)hexatrienyl)pyridinium dibromide; Invitrogen) and AM4-65 (Biotium). All dyes were used at a concentration of 10 μM diluted from a 500 μM stock solution in distilled water.

The confocal laser scanning microscopes (CLSM) used in this study were a Leica (Mannheim, Germany) TCS SP5 coupled to a DMI 6000B inverted microscope and a Zeiss (Jena, Germany) LSM 510 coupled to a Zeiss Axiovert inverted microscope. For the excitation of FM1-43FX and AM1-44, the 488 nm line of the argon laser was used, and the emitted fluorescence was detected in the range 505–550 or 560–615 nm. For the fluorescent dyes FM4-64 and AM4-65, excitation was performed at 514 nm and the fluorescent signal was detected between 660 and 720 nm. Images were taken using a 40x water immersion objective with a numerical aperture of 1.2 or a 63x water immersion objective with a numerical aperture of 1.4. All images included in this study are single optical sections and are positioned with vertical sides parallel to the long axes of the cells. The images in the time series (videos) are positioned with horizontal sides parallel to the long axes of cells.

Strong autofluorescence of compact chloroplast files hampered *in vivo* detection and counting of endosomal organelles stained by styryl dyes. Thus, *Chara* cells used in these experiments were locally irradiated with the blue light of a halide microscope lamp for 3 min at least 1 day prior to the experiments. This illumination resulted in a chloroplast-free window (area about 30,000 μm^2^ (ca. 200 × 150 μm wide rectangle) in which fluorescent endosomal organelles could be visualized. Data were collected from individual images with sizes between 35 × 35 and 55 × 55 μm of time series taken at intervals of at least 1.3 s. Care was taken to analyze non-overlapping regions of endoplasm. Therefore, the time distance between analyzed images varied between 1.3 and 2.6 s depending on the velocity of cytoplasmic streaming (Supplementary Videos 1, 2). Charasome number, size, charasome area fractions, and the number of endosomal particles per area were counted and analyzed using ImageJ (http://imagej.nih.gov/ij) and Sigma Plot 13 (Systat Software, San Jose, USA).

### Immunofluorescence

Fixation and staining for indirect immunofluorescence are described in detail in Schmoelzer et al. (2011). Cells were fixed in 1% (v/v) glutaraldehyde in 70% phosphate-buffered saline (PBS; 140 mM NaCl, 2.95 mM KCl, 2.38 mM KH_2_PO_4_, 7.61 mM Na_2_HPO_4_, 18.5 mM NaN_3_; pH 6.9) for 30 min at room temperature. Cells were dissected using a small pair of scissors at the onset of plasmolysis. Obtained fragments were further treated as follows: 3 × 15 min wash with PBS, 30 min treatment with 1 mg.ml^−1^ NaBH_4_ in PBS, 3 × 15 min wash with PBS, 30 min blocking with 1% (w/v) bovine serum albumin (BSA), and 50 mM glycine in PBS (blocking buffer), 2 h incubation with primary antibody anti-CHC (Agrisera AS10690; Vännäs, Sweden) at a concentration of 0.01 mg.ml^−1^; 3 × 30 min wash with PBS, overnight incubation at 4°C with secondary antibody, anti-rabbit-IgG Alexa 488 (Thermofisher, Waltham, USA), diluted 1:500 followed by 3 × 30 min wash with PBS. In order to visualize charasomes, cell fragments were stained after fixation with filipin III at a concentration of 3 mM for 5 min. After the final wash (3 × 5 min), cell fragments were mounted in PBS/glycerol 1:1. All antibodies were diluted in blocking buffer.

### Electron microscopy

Chemical fixation was performed as described by Foissner (1991). In brief, cells were fixed for 20 min at room temperature in 1% glutaraldehyde dissolved in phosphate buffer, pH 6.8. Following several washes in buffer, cells were postfixed overnight at 4°C in 2% OsO_4_ dissolved in buffer. After dehydration in an ethanol series at 4°C, cells were embedded in Agar low viscosity resin (Agar Scientific, Essex, Great Britain) via propylene oxide. Thin sections were stained with uranyl acetate and lead citrate.

Chemical fixation in the presence of filipin was performed according to Grebe et al. (2003) with slight modifications. Briefly, cells were fixed for 1 h at room temperature in 4% formaldehyde dissolved in 75% microtubule stabilizing buffer (50 mM Pipes, 5 mM EGTA, 5 mM MgSO_4_; pH 6.9) containing 0.5 mg.ml^−1^ (about 0.7 mM) filipin III. Glutaraldehyde was added to a concentration of 1%, and fixation was continued for 14 h at 5°C. After a wash in buffer, cells were postfixed for 2 h at room temperature in 1% OsO_4_ dissolved in distilled water containing 1.5% K_4_Fe(CN)_6_.3H_2_O. After dehydration through a graded ethanol series cells were embedded in Epon 812 (Agar Scientific) via propylene oxide.

Micrographs of ultrathin sections were taken using elastic bright-field mode with a LEO 912 transmission electron microscope, equipped with in-column energy filter (Zeiss, Oberkochen, Germany) and with a Philips 400 EM electron microscope (Eindhoven, the Netherlands).

### Cloning and sequence analyses

Total RNA was extracted from *C. australis* thalli, and transcriptomic data were obtained on the one hand from a normalized random primed cDNA library followed by 454 sequencing (Roche GS FLX system; EurofinsMWG) and on the other hand from a normalized cDNA library sequenced by Illumina Hiseq 2000 technology (BGI Genomics, Hongkong, China). Different annotated plant clathrin sequences were used for BLAST analyses (Altschul et al., 1990) in order to reveal putative *C. australis* clathrin cDNAs.

To verify these sequences, fresh total RNA was extracted from thalli of *C. australis* with TRI-Reagent according to manufacturer's instructions (Sigma Aldrich). Residual genomic DNA was digested using RNase-free DNase (EN0521, Thermo Fischer Scientific, Waltham, MA, USA), and first-strand cDNA was synthesized from 1 μg total RNA by M-MuLV Reverse Transcriptase (RevertAid; EP0441, Thermo Fischer Scientific) combined with an anchored oligo(d)T primer-mix according to the supplier's protocol. Obtained cDNA was used as template for PCR amplification with Phusion High-Fidelity DNA polymerase (F530S, Thermo Fischer Scientific), according to manufacturer's instructions. Due to sequence lengths, CHCs were divided into three sub-parts with an overlap of about 150 bp (for primers list see Supplementary Table 1), and reassembled *in silico* after sequencing. All PCR products were cloned into pJet1.2 cloning vector (K1231, Thermo Fischer Scientific) and sequenced. Accession numbers: CaCHC1 (KX555239), CaCHC2 (KX555240), CaCLC1a (KX951953), CaCLC1b (KX951954), CaCLC1c (KX951955), CaCLC2a (KX951956), CaCLC2b (KX951957).

The protein sequence alignments were performed by ClustalW (for alignment of two sequences) or Clustal Omega (for more than two sequences; EMBL-EBI). Conserved domains were detected using InterPro (Mitchell et al., 2015), and the conserved domain search tool of NCBI (Marchler-Bauer et al., 2015).

For Western blot analysis *C. australis* thalli were homogenized in liquid nitrogen using mortar and pestle. Ice cooled extraction buffer [2.5 volumes; 100 mM NaH_2_PO_4_ (pH 7.8), 100 mM KCl, 1 mM DTT, 0.04% Tween, and protease inhibitor cocktail (P9599; Sigma)] was added and the suspension was shaken on ice for 15 min, followed by centrifugation (15 min, 7500 × g, 4°C). The resulting supernatant was used for western blot analyses. Following separation on a 10% acrylamide separation gel, proteins were blotted onto a polyvinylidene difluoride membrane (PVDF; Merck, Darmstadt, Germany) for 75 min at 70 V. The membrane was blocked for 1 h in TBST-BSA (1%, w/v) at room temperature followed by addition of polyclonal CHC 1,2 antibody (Agrisera AS10 690, dilution 1:2000) and incubation for another hour with agitation. The PVDF membrane was washed three times for 5 min in TBST. The secondary antibody, a monoclonal anti-rabbit IgG coupled to alkaline phosphatase (Sigma-Aldrich), was used at a concentration of 1:20,000 in TBST (1 h). After another wash step (3 × 5 min in TBST), protein labeling was detected using CDP star detection kit (New England Biolabs, Ipswich, MA, USA) and a LAS 3000 mini imaging system (Fujifilm, Tokyo, Japan).

CHC protein sequences of *C. australis* were aligned with 61 CHC sequences obtained from NCBI GenBank of diverse species of higher plants, ferns, mosses, fungi, and green algae using the program Geneious 6.1.2 created by Biomatters. The web-portal Alter (Alignment Transformation Environment) was applied to convert the alignment in FASTA format into a Nexus-file. Then, the dataset was analyzed with the program ProtTest2.4 (Abascal et al., 2005) using Akaiko Information Criterion (AIC) scores to achieve the best model for phylogenetic analysis. The analysis was computed on Cipres Science Gateway V.3.1 (Miller et al., 2010) using BEAST 1.8.2 (Drummond et al., 2012) on Xsede, and on the basis of the LG+G+I model. Five parallel analyses were run under a lognormal relaxed clock for 10,000,000 chains, and every 1000^th^ tree was sampled. The five obtained log-files were controlled by the program Tracer v1.5, if the analyses were suitable for final phylogenetic tree configuration. The tree-files with branch lengths of each run were combined to one common tree-file, using LogCombiner1.8.2 in BEAST. Finally, this file was used to summarize the sampled trees with a burn-in of 20% (10,000 trees) to a maximum clade credibility tree by TreeAnnotator1.8.2 in BEAST. The dataset was also analyzed by ML bootstrapping in RAxML, with the graphical front-end raxmlGUI 1.3 using 1000 replicates. All trees were illustrated using the program FigTree v1.3.1.

## Results

### Prolonged darkness, decreases size, and abundance of charasomes

**Figure 1:**
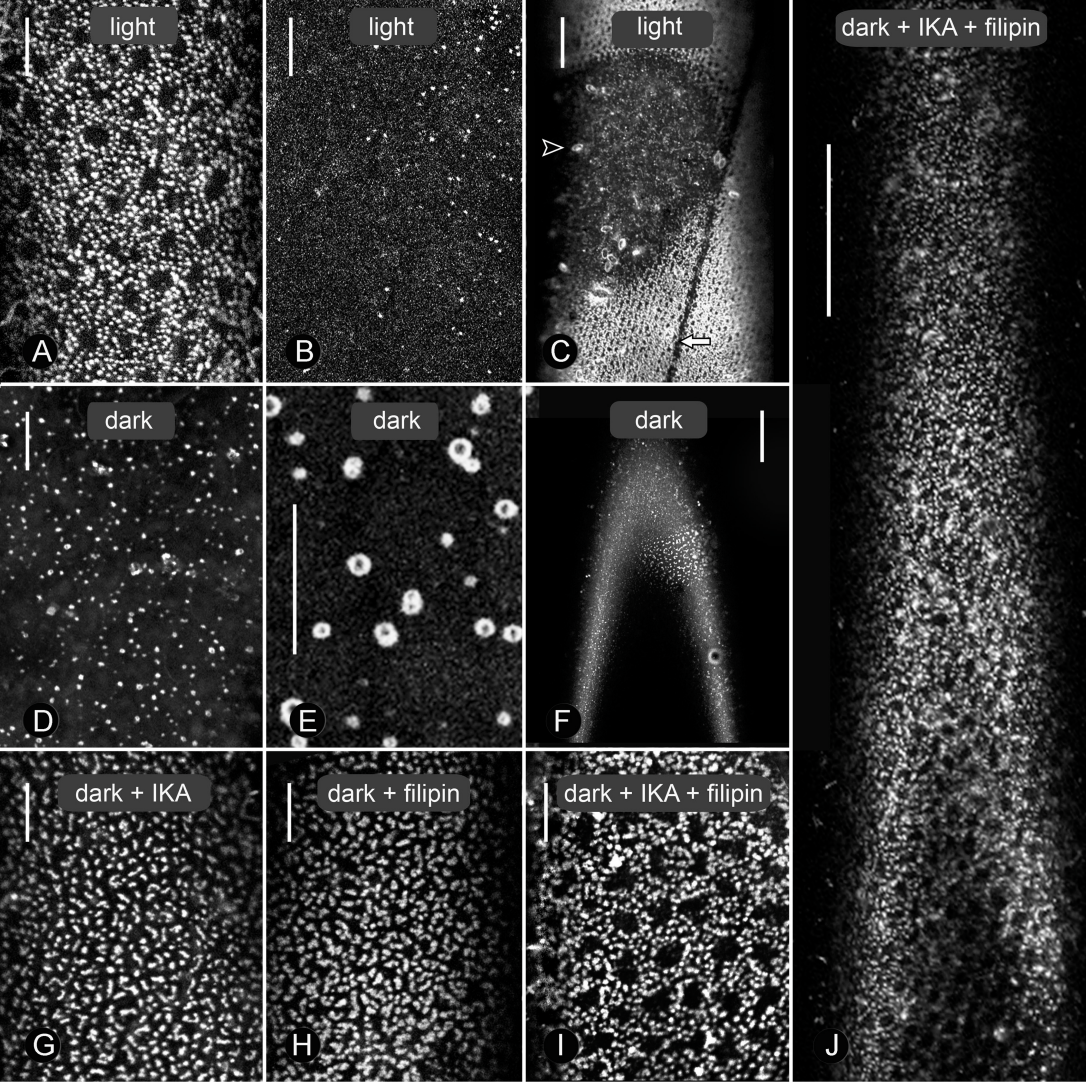
**Effect of dark-incubation and inhibitor treatments on size and distribution of charasomes stained by fluorescent FM1-43 in branchlet internodal cells of *Chara australis***. Under standard conditions of 14/10 h light/darkness, extended charasome-rich areas **(A)** alternate with small, charasome-poor or -free regions (**B**, and smaller magnification **C**). The white arrow in **(C)** indicates the neutral line, while the arrow head points to an FM-stained epiphyte. Panels **(D)** and **(E)** show different magnifications of cells exposed to continuous darkness for 12 days (control for **G–J**). Charasomes are mostly small and uniformly distributed **(D)**, but “islands” with larger charasomes occasionally remained **(F)**. **(G–J)** Effect of 1 μM ikarugamycin (IKA; **G**), 1.5 μM filipin **(H)**, and of a combination of both inhibitors **(I, J)** on dark-treated cells. Note the extended charasome-rich regions **(J)**. Bars are 10 μm **(A,B,D,E,G–I)** and 50 μm **(C,F,J)**.

In contrast to formation, charasome degradation has not been described previously in sufficient detail. During the course of this study, we used CLSM and electron microscopy to investigate charasome degradation induced by dark incubation. Before dark incubation, branchlet internodal cells were isolated from the thallus and exposed to standard light/dark conditions for 8 days. The distribution of charasomes in such a cell is revealed after staining with fluorescent plasma membrane markers and analysis in the CLSM. Figures 1A–C show that extended charasome-rich regions (Figure 1A and brighter cell parts in Figure 1C) alternate with charasome-poor areas (Figure 1B and darker cell parts in Figure 1C; compare Schmoelzer et al., 2011). The maximum charasome area fraction in these cells, i.e., the cell surface area occupied by charasomes, was up to 48%, with median values between 30 and 40% (see Schmoelzer et al., 2011; Sommer et al., 2015). When these cells were incubated in continuous darkness, size, and number of charasomes decreased significantly (Figure 1D, Supplementary Figure 1), and the remaining, small charasomes often showed a ring-like, “hollow” appearance (compare Figure 1E). Furthermore, the charasome area fraction along the cell surface became more homogeneous. Occasionally, small islands with high charasome area fractions remained, indicating that charasome degradation did not occur uniformly within one cell (compare Figure 1F). The extent to which charasomes were degraded varied according to the original charasome area fraction and the length of dark incubation. In any case, after 12 days of continuous darkness, the median value for maximum charasome area fraction per cell decreased significantly to 10% or less. Dark-treated internodal cells retained continuous cytoplasmic streaming as shown in Supplementary Video 1 and were able to form numerous and large charasomes again, when exposed to standard light/dark conditions (not shown; compare for instance to Lucas and Franceschi, 1981; Foissner et al., 2015).

### Coated vesicles are involved in charasome degradation

**Figure 2:**
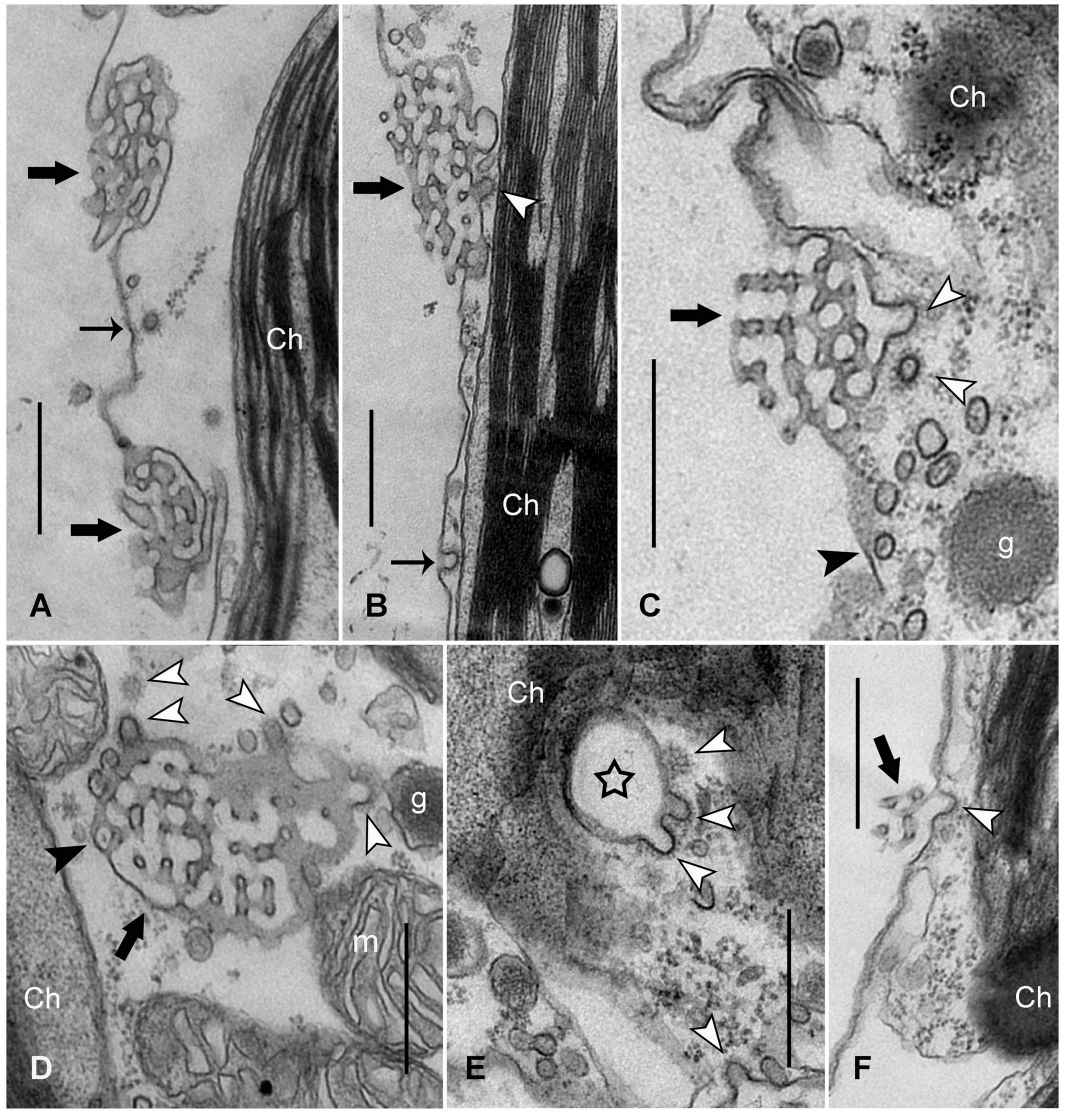
**Fine structure of charasomes in a control cell (A)** and in cells exposed to continuous darkness for 3 days **(B–F)**. **(A)** Longitudinally sectioned small charasomes (thick black arrows) in a cell exposed to standard light/dark conditions. CV close to a nascent CP (thin black arrow) is seen at the plasma membrane (Ch, chloroplast). **(B,C)** Longitudinal sections of dark-incubated cells: CPs and CVs (white arrow heads) are detectable at charasomes (thick black arrows). The thin black arrow indicates a CP at the smooth plasma membrane. Note: there are numerous smooth vesicles near the charasome, some of them containing a central core (black arrow head in **C**) (g, glycosome). **(D)** Numerous CPs and CVs (white arrow heads) are present at this tangentially sectioned charasome (thick black arrow). The black arrow head points to a charasome tubule containing a central core. (m, mitochondrion) **(E,F)** Late stages of darkness-induced charasome degradation. CPs (white arrow heads) formed at the surface of a tangentially sectioned charasome (star in **E**; note absence of inner tubules) and at a longitudinally sectioned charasome (thick black arrow in **F**). Note the characteristic polygonal lattice of the clathrin coat in **(D,E)** (upper white arrow heads). Bars are 500 nm.

These findings prompted us to inspect the fine structure of dark-exposed cells, in order to decipher the mechanism(s) of charasome degradation. Figure 2A shows an electron micrograph of a branchlet internodal cell, exposed to standard light/dark conditions. The charasomes consist of a complex meshwork of anastomosing tubules formed by plasma membrane invaginations. CPs and CVs with an average inner diameter of 65.7 ± 11.0 nm (*n* = 77) were occasionally seen at the smooth plasma membrane between charasomes, but rarely at the cytoplasmic surface of charasomes, indicating that these organelles were no longer growing and can thus be called “mature” (compare Lucas and Franceschi, 1981; Pesacreta and Lucas, 1984; Sommer et al., 2015). Charasomes of cells exposed for 1 or 2 days to continuous darkness looked similar to control cells. However, after 3 days the degradation was clearly visible, and we detected up to four CPs per thin section at the inner surface of charasomes (Figures 2B–F). These CPs eventually pinched off as CVs with an inner diameter between 51.4 and 110.9 nm (70.9 ± 11.7 nm on average, *n* = 30), as suggested by their presence in the immediate surroundings of the CPs (Figures 2C,D, white arrowheads). The morphology of this coat showed striking similarity to the typical clathrin lattice. The average size of clathrin CVs measured in the present study (around 60–70 nm) is within the range commonly reported in the literature: 50–60 nm in *Chara* (Lucas and Franceschi, 1981), 84–91 nm in carrot cells (Coleman et al., 1987), 80 nm in pea cotyledons (Harley and Beevers, 1989), 60–100 nm in epithelial cells (Bonifacino and Lippincott-Schwartz, 2003). However, our values are larger than the size of CVs found by Limbach et al. (2008) in the apex of *Chara* rhizoids (30 nm). It is likely that these differences in size are due to different protocols used for the preparation of electron microscopy samples (e.g., chemical fixation vs. high pressure freezing and cryo-substitution). Abundance of CPs and CVs varied between cells, cell fragments, and even between charasomes within one fragment. This is consistent with results obtained from AM4-65/FM1-43FX-*in vivo* staining, showing that charasome degradation does not occur uniformly within one cell (Figure 1F). Bubble-like charasomes with few or no inner tubules were characteristic for late stages of charasome degradation (Figure 2E), and perhaps corresponded to the “hollow” charasomes observed with CLSM (Figure 1E).

### Charasome degradation is clathrin-dependent

The morphology and size of CPs and CVs involved in darkness-induced degradation of charasomes strongly suggested the presence of clathrin polymers as building-blocks of the coat (see characteristic polygonal lattice in Figures 2D,E, upper white arrrow heads). In order to prove this, we applied IKA, an inhibitor considered to be specific for clathrin-dependent endocytosis (Hasumi et al., 1992; Luo et al., 2001; Moscatelli et al., 2007; Onelli et al., 2008; Bandmann et al., 2011; Elkin et al., 2016).

As plasma membrane recycling may also occur via a clathrin-coat independent mechanism, we tried to dissect the relative contribution of these two pathways to the process of charasome dismantling. There is convincing evidence that both pathways require the participation of sterol-like substances (e.g., Grebe et al., 2003; Baral et al., 2015). Thus, inhibition of endocytosis by sterol depletion would affect both mechanisms. If the effect of sterol depletion on charasome degradation would significantly exceed the inhibition achieved by clathrin-coat inhibitors, it would follow that a clathrin-independent pathway is operative. We therefore used filipin, a sterol complexing fluorochrome (Boutté et al., 2011) and methyl-ß-cyclodextrin (MβCD), a non-fluorescent sterol binding agent (Ilangumaran and Hoessli, 1998) for our investigations.

**Figure 3:**
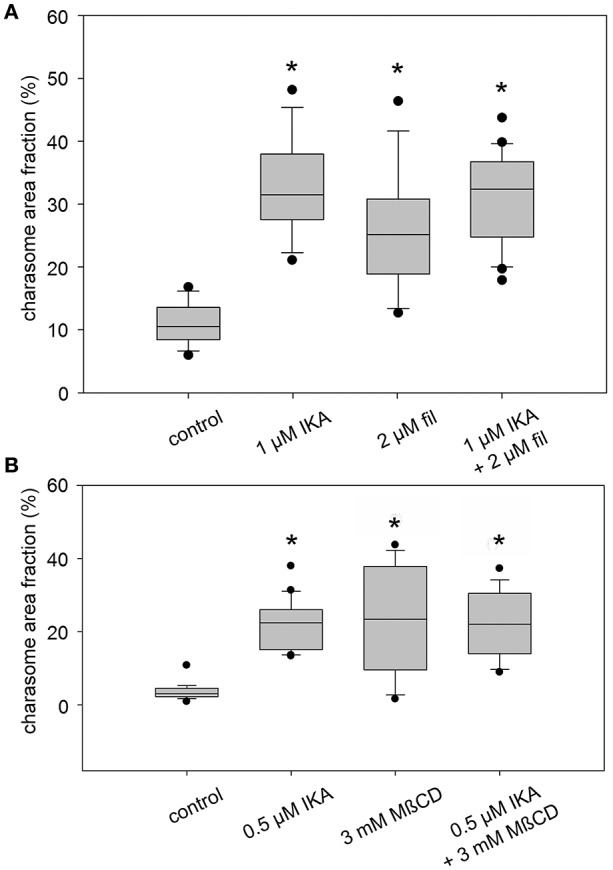
**Inhibitory effect of ikarugamycin (IKA), filipin (fil), and methyl-β-cyclodextrin (MβCD) on darkness-induced charasome degradation**. Cells were dark-treated in solvent-only or inhibitor solutions for 12 days. Charasome area fraction (% of cell surface area occupied by charasomes) was measured after staining with FM1-43 or AM1-44. **(A)** Effect of IKA, filipin, and a combination of both inhibitors on charasome area fraction of dark-treated cells. **(B)** Effect of IKA, MßCD, and a combination of both inhibitors on charasome area fraction of dark-treated cells. Box-and-whisker plots show median values with upper and lower quartiles (boxes), whiskers indicate 10^th^ and 90^th^ percentiles and outliers (dots) with *n* (number of cells) = between 14 and 22. Differences between median values of control and treated cells are significant (asterisks; one way analysis of variance, *P* ≤ 0.001).

The effects of IKA, filipin, and MßCD on dark-induced degradation of charasomes were tested separately, as well as in combination. We found that cells exposed to darkness for 9–13 days and simultaneously treated with 0.5 or 1 μM IKA, had significantly higher maximum charasome area fractions than the solvent treated cells (Figures 1G, 3A,B). Hence, IKA significantly delayed, but did not fully arrest, charasome degradation. The same result was detected using 1.5 μM filipin (Figure 1H), as well as a combination of both inhibitors (Figures 1I,J, 3A). Thus, all three treatments significantly delayed dark-induced charasome degradation, but there was no additive effect indicating insignificant contributions from clathrin-independent endocytic pathways.

Similar results were obtained when MβCD was used as a sterol-complexing agent (Figure 3B). In cells incubated in darkness for 9–13 days, 2–3 mM MßCD significantly inhibited charasome degradation. Again, the combination of IKA and MßCD showed no additive effect, reinforcing the idea that charasome degradation follows mainly a clathrin-dependent pathway. While in dark-incubated cells without simultaneous inhibitor treatment, the high charasome area fractions were restricted to small “islands” (Figure 1F), in inhibitor-treated cells these areas extended over several 100 μm (compare Figure 1J).

It is important to mention that cells treated with up to 1 μM IKA (Supplementary Video 1), 2 μM filipin, 3 mM MßCD, or with a combination of one of the sterol complexing agents with IKA for 1 to 2 weeks, still internalized styryl dyes. Thus, long-term treatment with low concentrations had no arresting effect on constitutive endocytosis.

**Figure 4:**
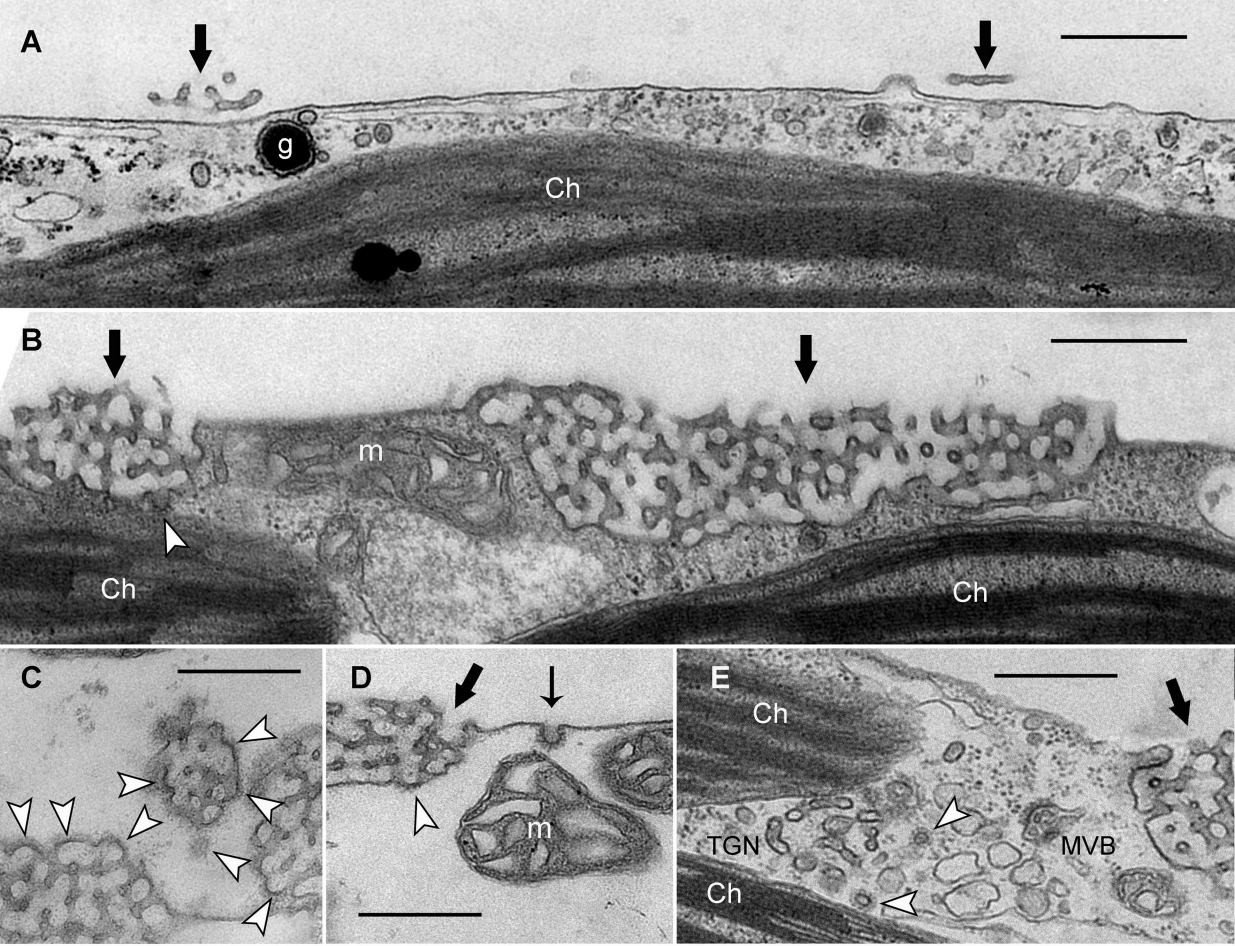
**Electron micrographs of branchlet internodal cells treated with solvent (A)** or ikarugamycin **(B–E)**. **(A)** Smooth plasma membrane in a cell dark-incubated for 6 days in the presence of 0.05% DMSO (control condition). Thick black arrows indicate remnants of charasomes. (Ch, chloroplast; g, glycosome). **(B–D)** Cortical cytoplasm in cells dark-incubated for 6 days in the presence of 0.5 μM ikarugamycin (IKA). Large charasomes (thick black arrows) and CPs or CVs (white arrow heads) at the charasomal surface. Typical CPs are present at the smooth plasma membrane (thin black arrow in **D**) (m, mitochondrion). **(E)** No effect of IKA (4 μM for 30 min) on the formation of CVs (arrow heads) from the TGN (MVB, multivesicular body). Bars are 500 nm.

Next, we investigated the fine structure of IKA-treated cells, in order to find out whether or not IKA inhibits formation of clathrin coats. Figure 4A shows an electron micrograph of a control experiment in which a branchlet internodal cell was dark-incubated for 6 days in the presence of DMSO (0.05%). Due to prolonged darkness, charasomes were degraded until only smooth plasma membrane and a few charasome remnants could be detected (black arrows in Figure 4A). In the presence of 0.5 μM IKA large charasomes (black arrows in Figure 4B) were still detectable even after 6 days of darkness, showing the inhibitory effect of IKA on degradation of charasomes. IKA, however, did not inhibit the formation of CPs at the surface of charasomes (arrow heads in Figures 4B–D). CVs were occasionally seen in the immediate neighborhood of charasomes. CPs (thin arrow in Figure 4D) and CVs were also present at smooth plasma membrane regions, indicating ongoing constitutive endocytosis at the low IKA concentrations, used for long-time treatments. The average inner diameter of these vesicles was between 50.9 and 82.9 nm, well in the range of the controls (see above). These findings are consistent with the uptake of styryl dyes (Supplementary Video 1). Furthermore, we detected CPs and CVs at the TGN (arrow heads in Figure 4E) in cells treated with IKA up to a concentration of 4 μM.

We further studied the fine structure of cells treated with the sterol complexing dye filipin. Supplementary Figure 2 shows the effect of filipin on the cortical cytoplasm of *Chara* internodes. After 30 min incubation in 1.5 μM filipin, followed by conventional fixation of cells for electron microscopy, plasma membrane, charasome tubules, and membranes of other organelles had a fuzzy appearance (Supplementary Figure 2A; compare to Grebe et al., 2003). Similar images were obtained when cells were not pretreated with filipin but fixed in the presence of filipin (Supplementary Figure 2B). In both cases, charasomes and plasma membrane appeared to be more affected than the membrane of mitochondria and chloroplasts. This may indicate higher concentrations of sterols in these compartments. Neither CPs nor CVs could be identified on images taken from thin sections of filipin-treated or filipin-fixed material.

**Figure 5:**
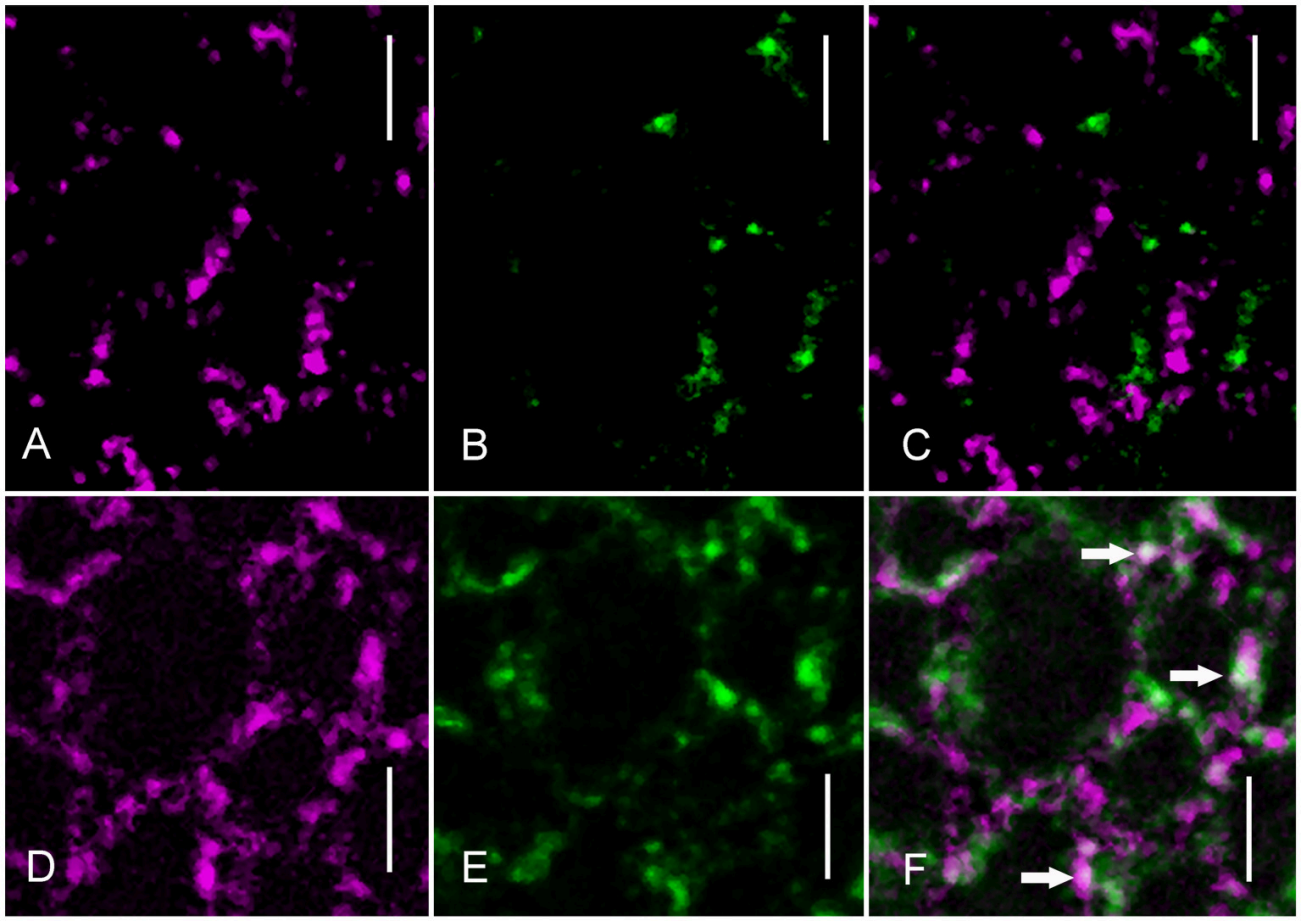
**Anti-clathrin immunofluorescence (green) and distribution of filipin-stained charasomes (magenta). (A–C)** Charasomes do not overlap with clathrin epitopes in a cell under standard light/dark conditions (**C** is the merged image). **(D–F)** Colocalizations of charasomes and clathrin-positive regions (arrows in the merged image **F**) are present in response to dark-incubation for 3 days. Bars are 5 μm.

The participation of clathrin in charasome degradation was further supported by experiments using immunofluorescence and an antibody against CHCs. Schmoelzer and co-worker identified filipin as a suitable fluorescent marker for charasomes in 2011. Thus, we used it in this study to visualize charasomes after immunolabeling. Under standard light/dark conditions, the distribution of charasomes is independent of the regions recognized by anti-clathrin (Figures 5A–C). However, after 3 days of incubation in darkness, colocalizations of filipin-stained charasomes and clathrin-positive patches were frequently observed (Figures 5D–F). Scatter plots and correlation coefficients for both treatments are shown in Supplementary Figure 3.

### Effect of inhibitors on constitutive endocytosis in *Chara* internodal cells

The data presented in this study so far, showed that low concentrations of IKA inhibited degradation of charasomes. Nonetheless, CPs and CVs were still present, especially at the smooth plasma membrane. We therefore investigated the effect of IKA and MßCD on internalization of styryl dyes, which presumably occurs via constitutive endocytosis (clathrin-dependent and/or independent) from smooth plasma membrane regions.

**Figure 6:**
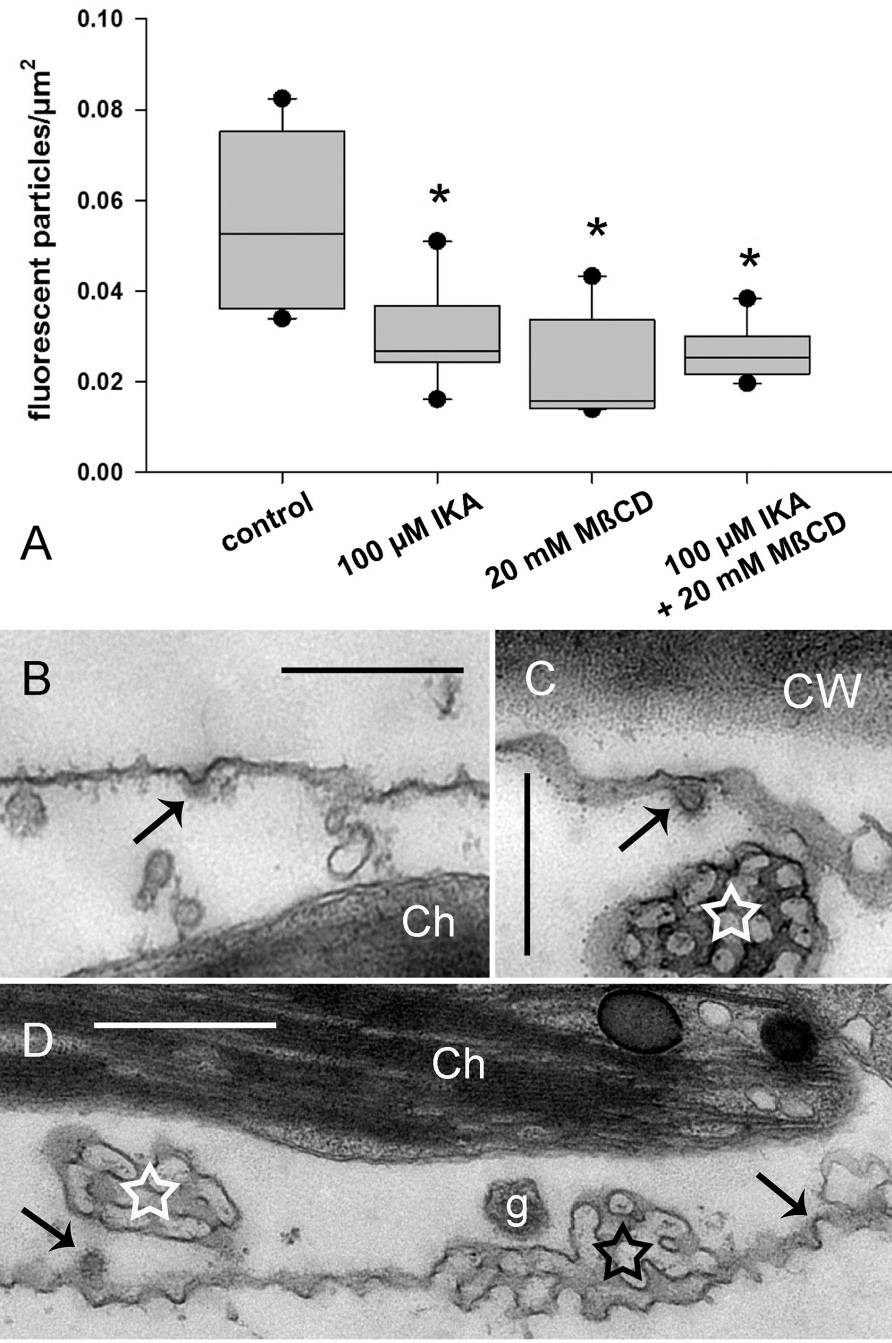
**Effect of ikarugamycin (IKA) and methyl-ß-cyclodextrin (MßCD) on internalization of AM1-44 in internodal cells of *Chara australis*. (A)** Relative numbers of AM1-44-stained organelles in control cells, in cells treated with 100 μM IKA, 20 mM MßCD, and in cells treated with both inhibitors simultaneously. Cells were inhibitor- or solvent (DMSO)-treated for 30 min, pulse-labeled for 2 min, and chased within the next 30 min in inhibitor- or DMSO-containing artificial fresh water. The number of fluorescent particles was analyzed within 30 min. Box-and-whisker plots show median values with upper and lower quartiles (boxes), dots indicate outliers, and whiskers indicate 10^th^ and 90^th^ percentiles; *n* (number of cells) = 5–7. Differences between median values of control and treated cells are significant (asterisks; *t*-test, *P* ≤ 0.002). **(B,D)** Electron micrographs of cells treated with 100 μM IKA for 90 min. CPs and CVs (arrows) are present at the smooth plasma membrane. Ch, chloroplast; CW, cell wall; g, glycosome. **(C)** Electron micrograph of a cell treated with 8 μM IKA for 12 h. White stars indicate tangentially sectioned charasomes and black asterisk indicates longitudinally sectioned charasome. Bars are 500 nm.

Figure 6A shows that IKA, MßCD, and a combined treatment significantly reduced, but did not arrest, uptake of fluorescent markers (Figure 6A; Supplementary Video 2). Accordingly, electron microscopy showed CPs and CVs at the smooth plasma membrane (Figures 6B–D). Notably, the average size (inner diameter) of the clathrin CVs in cells treated with 100 μM IKA for 90 min was significantly lower (49.6 ± 8.76 nm, *n* = 45, *P* < 0.001) than in control samples. It has to be stressed, that much higher concentrations were required to affect constitutive endocytosis than charasome degradation. In the case of IKA, significant effects on constitutive endocytosis were observed at 100 μM or higher. When compared to the IKA concentrations necessary for inhibition of charasome degradation in long-time treatment, this corresponds to a 100- or 200-fold increase. In the case of MßCD, 20 mM were required. This corresponds to a 7- to 10-fold increase in inhibitor concentration. As observed in the charasome degradation experiments, a combined treatment of clathrin inhibitor IKA and sterol complexing MßCD had no additive effect on internalization of styryl dyes. In spite of these high inhibitor concentrations, cells retained constant and steady cytoplasmic streaming (Supplementary Video 2 for IKA-treated cells).

### Identification, cloning, and phylogenetic analysis of clathrin proteins in *Chara australis*

The data presented above indicate a central role of clathrin in charasome degradation. This encouraged us to search for clathrin homologs in the transcriptome of *Chara*. Databases of *C. australis* sequences were created using 454 as well as Illumina RNA sequencing. These databases were searched for clathrin-like sequences. The obtained results revealed homologous proteins of CHC as well as CLC.

**Figure 7:**
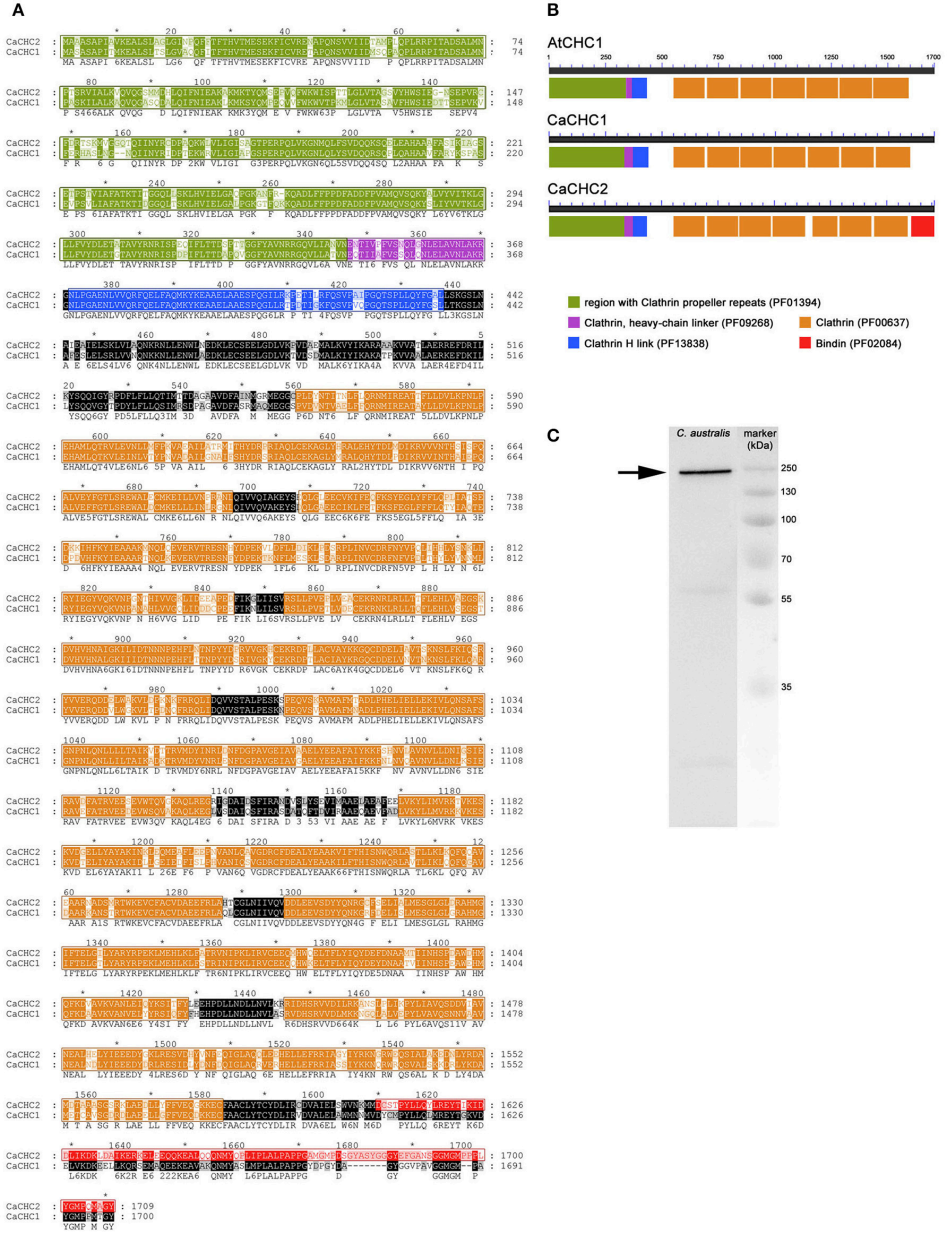
**Clathrin heavy chain proteins in *Chara australis*. (A)** Protein sequence alignment of clathrin heavy chain (CaCHC1 and CaCHC2) proteins from *Chara australis* performed with ClustalW. Identical residues are highlighted in black. Numbering of amino acid residues begins at the first methionine. **(B)** Arrangement of domains on CHC proteins: AtCHC1 (*Arabisopsis thaliana*); CaCHC1, CaCHC2 (*C. australis*). Conserved domains are displayed in different colors as indicated. Pfam database classifications of annotated domains are given in brackets. Note the unique bindin-like domain on CaCHC2. **(C)** Western blot of *C. australis* protein extract. The prominent CaCHC band (arrow) was visualized using an antibody against plant CHCs. Marker = molecular mass marker.

Two heavy chains were cloned, sequenced, and named CaCHC1 (1700 amino acids, calculated MW of 192.4 kDa; accession number KX555239) and CaCHC2 (1709 amino acids, calculated MW of 193.6 kDa; accession number KX555240). While the two well known CHC proteins of *Arabidopsis thaliana* (AtCHC1: At3g11130, accession number Q0WNJ6; AtCHC2: At3g08530, accession number Q0WLB5) showed 97% sequence homology when compared to each other, the two CHCs of *C. australis* shared only 75% identity (see Figure 7A). Furthermore, CaCHC1 showed higher sequence similarities to CHC of all other plant species investigated than CaCHC2. For example, compared to *A. thaliana*, CaCHC1 revealed 79% homology to AtCHC1 as well as AtCHC2, while CaCHC2 showed only 73%. Figure 7B illustrates the arrangement of conserved domains along the amino acid sequences. AtCHC1 protein is shown as example of known plant CHCs. The arrangement of domains on CaCHC proteins is comparable to AtCHC. However, CaCHC2 showed a specialty at the C-terminus: a weak but measurable similarity with a bindin domain was detected on the last about 100 amino acids.

Moreover, CHC proteins were visualized by western blot analyses in *C. australis* protein extracts. The polyclonal CHC 1,2 antibody (Agrisera AS10 690) was used (Figure 7C) for detection. A prominent band was visible slightly below marker band 250 kDa, as expected from the calculated molecular mass of the proteins of about 193 kDa.

**Figure 8:**
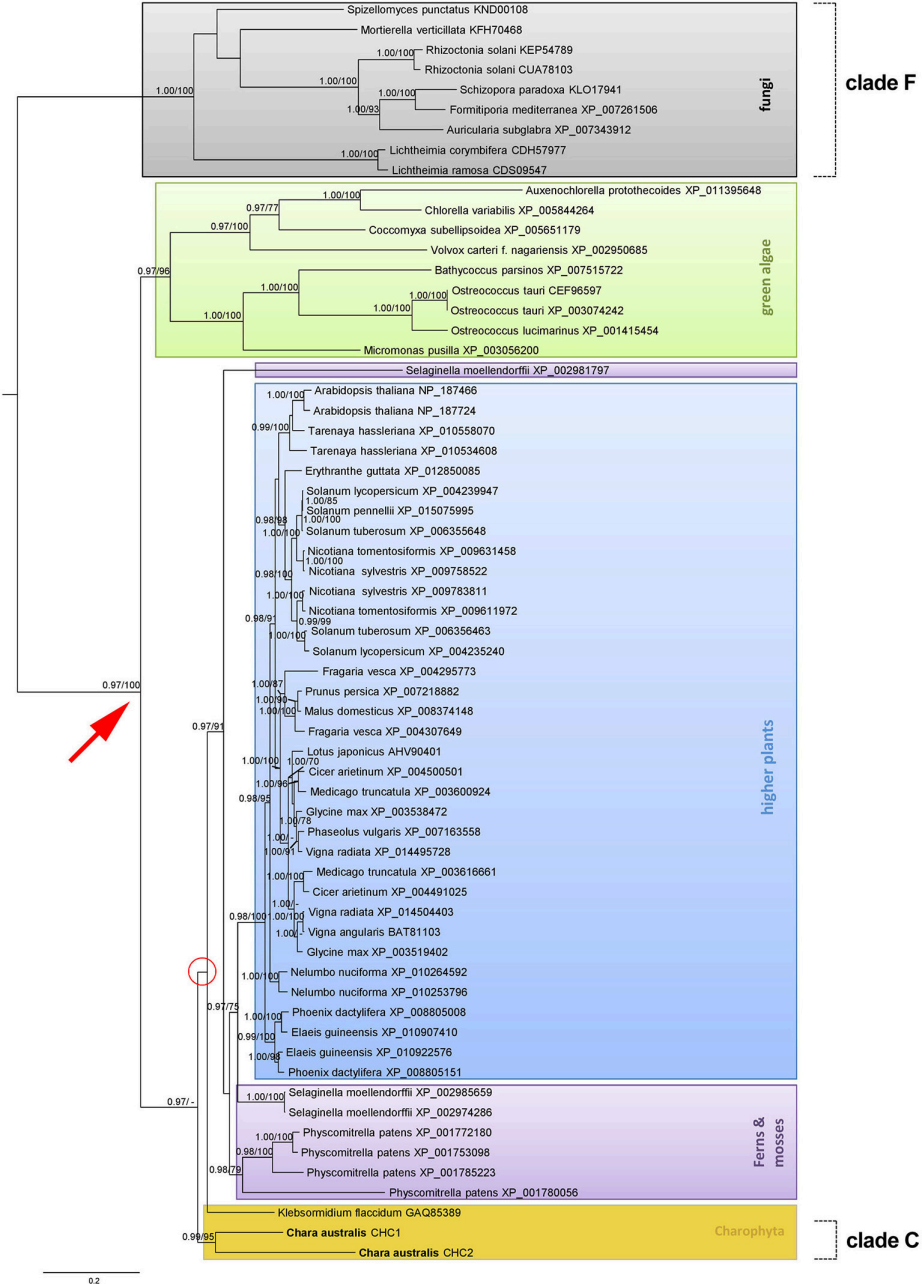
**Phylogenetic analysis of different clathrin heavy chain protein sequences**. Maximum clade credibility tree of different CHCs calculated with BEAST. Considered as strongly supported are branches with posterior probabilities (PP) ≥ 0.95% reflecting the posterior median node heights for the clades, and ML bootstrap support (ML B) ≥ 70%. The bar indicates substitutions per site. Different groups of species origin are highlighted in color.

In order to classify CaCHC proteins under the family of CHCs, phylogenetic analyses were performed. As shown in Figure 8, the 63 aligned CHC protein sequences were divided into several major groups: fungi, green algae, Charophyta, ferns and mosses, and higher plants. Fungal CHC proteins formed a separate clade (clade F), and therefore presented the outgroup in this phylogenetic analysis. The plant CHC sequences were divided into two major groups (red arrow in Figure 8), where the green algae CHCs were sister to the group of Charophyta, as well as all other plant sequences. Clade C (containing CaCHC1 and CaCHC2) was combined with groups including land plant sequences (ferns, mosses, and higher plants), instead of being classified into the group of other green algae (green box in Figure 8). Interestingly, the CHC protein sequence of *Klebsormidium flaccidum* did not cluster within clade C, but showed sister relations to both sequences of *C. australis* and other land plants.

**Figure 9:**
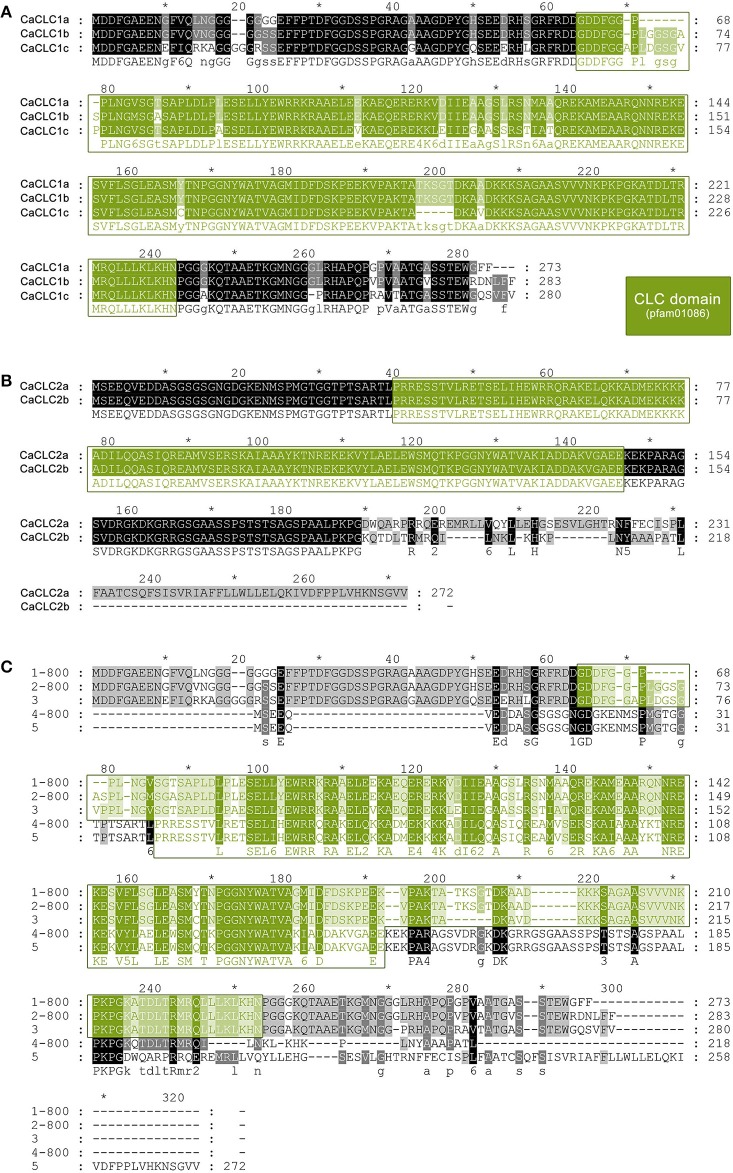
**Protein sequence alignment of clathrin light chain proteins from *Chara australis*.** Protein sequence alignments of CaCLC1 and CaCLC2 variants were performed with ClustalW (for two sequences) and ClustalOmega (for more than two sequences). Panel **(A)** shows the alignment of CaCLC1 variants, Panel **(B)** shows the alignment of CaCLC2 variants, and the alignment of all variants is displayed in **(C)**. Identical residues are highlighted in black and the conserved clathrin light chain domain (pfam01086) is displayed in green. Numbering of amino acid residues begins at the first methionine.

In addition to CHC proteins we also had a look at CaCLCs. Several CaCLC sequences were detected, cloned, and sequenced. Protein sequence alignments revealed two groups of highly similar proteins: CaCLC1 and CaCLC2. CaCLC1 comprises three expressed variants: CaCLC1a (273 amino acids, calculated MW of 28.8 kDa; accession number KX951953), CaCLC1b (283 amino acids, calculated MW of 29.9 kDa; accession number KX951954), and CaCLC1c (280 amino acids, calculated MW of 29.7 kDa; accession number KX951955). CaCLC2 comprises two expressed variants: CaCLC2a (272 amino acids, calculated MW of 30.0 kDa; accession number KX951956) and CaCLC2b (218 amino acids, calculated MW of 23.6 kDa; accession number KX951957). Protein sequence alignments of all CaCLCs are shown in Figure 9. CLC proteins are variable in their amino acid composition. The three *A. thaliana* CLCs (AtCLC) revealed only 37–49% protein sequence homology when compared to each other, while CaCLCs shared 26–93% homology. CaCLC1 proteins shared 25–32% similarity with AtCLCs and 24–28% to *Selaginella moellendorfii* CLCs. The two CaCLC2 proteins possessed 71% of identical amino acids and share 22–26% similarity to *A. thaliana* CLCs and 21–25% to *S. moellendorfii*. Amino acid sequence identities were calculated with the bioinformatic software CloneManager (http://www.scied.com/).

## Discussion

Membrane trafficking in plant cells is a matter of intensive study and consistent details about this intricate and highly orchestrated process are continuously revealed. In contrast, comparatively little attention was given to deciphering the routes and identifying the main players in endocytosis/exocytosis events in algal cells. Our group was hitherto successful in detecting and characterizing some of the key regulators of endocytosis in the green alga *C. australis*. Thus, we identified two members of the RAB5 GTPase family, CaARA6 (Hoepflinger et al., 2013) and CaARA7 (Hoepflinger et al., 2015) together with one of their putative interaction partners, a member of the SNARE- family (CaVAMP72, see Hoepflinger et al., 2014). Recently, hints inferred from our work with the inhibitor wortmannin (Foissner et al., 2016) suggested the role of other endocytosis modulators in *Chara*, the PIP kinases. In the present investigation, we focused on another crucial molecule in cellular membrane trafficking, clathrin, and we highlighted its role in constitutive endocytosis and plasma membrane shaping, with particular reference to charasomes.

### Central role of clathrin in charasome degradation and formation

The increase of plasma membrane area is a widespread strategy to achieve high rates of transport. Higher plant cells irreversibly form secondary wall ingrowths in order to amplify the plasma membrane surface area and to accommodate appropriate transporters. These so-called transfer cells are considered to play an important role in nutrient distribution and, in water plants, carbon uptake (Offler et al., 2003; Ligrone et al., 2011). Characean algae build structured elaborations of plasma membrane, which can be degraded and re-established according to the needs of the cell. Here, we used electron microscopy, immunofluorescence and inhibitor studies, combined with quantitative live cell imaging, to dissect the role of clathrin in degradation of charasomes. We found that dark incubation of *Chara* internodal cells causes the appearance of numerous CPs at mature charasomes and their release as CVs. This leads to a decrease in size and, eventually, to the disappearance of these organelles. Electron micrographs revealed the presence of the characteristic polygonal lattice of clathrin coat. Furthermore, our experiments with IKA, a specific inhibitor of clathrin-dependent endocytosis, as well as immunofluorescence with an antibody against CHCs, confirmed that the membrane coat consists of clathrin and that clathrin is involved in charasome degradation.

Clathrin most probably also plays a central role in charasome formation, which was studied in detail by Lucas and co-workers (Franceschi and Lucas, 1981; Lucas and Franceschi, 1981). These authors showed that numerous CPs (although the coat was not explicitly denominated as clathrin) are present at the surface of growing charasomes. According to their presumed developmental sequence, these coated regions are not released as CVs, but instead are involved in the fusion of membranes into charasome tubules.

Our recent work described the effects of wortmannin, an inhibitor of PIP3 and PIP4 kinases, in characean internodal cells. Wortmannin not only affects the morphology of multivesicular bodies, as known from other cells, but also inhibits clathrin dependent release of vesicles from the TGN and promotes its transformation into a complex tubular network which has a striking resemblance to charasomes (Foissner et al., 2016). This is consistent with observations that charasome membranes are delivered by TGN-derived vesicles. The effect of wortmannin on the TGN perhaps also indicates that PIP3 and/or PIP4 kinases are directly or indirectly involved in charasome formation and degradation.

Under steady state conditions, i.e., at non-growing and at non-degrading charasomes, the clathrin coats are largely absent, suggesting low membrane turnover. This is probably the reason why charasomes remain stained up to several days, after pulse-labeling with endocytic markers such as styryl dyes.

### IKA does not inhibit clathrin coat formation but affects the release of vesicles

The antiprotozoan antibiotic IKA (Jomon et al., 1972) was introduced as an inhibitor for clathrin-dependent endocytosis (Hasumi et al., 1992; Luo et al., 2001) in murine and human cells on the basis of isotope and biochemical measurements. The substance was later used to dissect endocytic pathways in plant cells (Moscatelli et al., 2007; Onelli et al., 2008; Bandmann and Homann, 2012; Nagawa et al., 2012). In all these studies, IKA significantly decreased endocytosis, although the precise action of IKA remained obscure. In this respect, it would be interesting to know, whether IKA inhibits the formation of a clathrin coat or acts on later steps of endocytosis. Unfortunately, the quality of the hitherto published electron micrographs of IKA-treated plant cells does not allow the identification of a (clathrin) coat (Moscatelli et al., 2007; Onelli et al., 2008). However, immunostaining of pollen tubes suggested that IKA could increase the association of CHCs with the plasma membrane and affect clathrin distribution, possibly by inhibiting the pinching off of clathrin-CVs (Moscatelli et al., 2007). Our images show for the first time, that CPs are abundant at the smooth plasma membrane and, especially, at degrading charasomes of IKA-treated cells. This adds support to the hypothesis that IKA does not inhibit the formation of the clathrin coat. More probably, it causes an inhibition or delay of a release of CVs. A possible scenario suggests that the clathrin coat becomes stabilized by IKA, so that dynamin or other proteins involved in the pinch off, cannot adequately bind. This stabilization could also account for the significantly lower diameter of CVs formed in the presence of 100 μM IKA. Consistent with our results, Elkin and co-workers recently also found that IKA appears to block the maturation and/or pinching off of CVs from the plasma membrane (Elkin et al., 2016).

IKA is a potential candidate for cancer therapy and has been reported to induce apoptosis in leukemia cells via increasing the cytoplasmic Ca^2+^ (Popescu et al., 2011 and references therein). *Chara* internodal cells, however, survive several weeks' treatment at 1–2 μM, which are immediately toxic to animal and human cells. Even at higher concentrations (up to 150 μM), internodal cells continued steady cytoplasmic streaming (Supplementary Video 2), which is a valuable indicator of cell viability in characean algae. This suggests that IKA has a very specific effect on clathrin-dependent endocytosis in *Chara* internodal cells.

Interestingly, much higher concentrations of IKA had to be used for inhibition of constitutive endocytosis than for charasome degradation. Concentrations required to inhibit charasome degradation were in the range between 0.5 and 1 μM. Slightly higher concentrations (3–5 μM) were used to inhibit uptake of nanogold and FM-dyes in pollen tubes (Moscatelli et al., 2007; Onelli et al., 2008), and to reduce the internalization of PIN1-GFP in tobacco epidermal cells (Nagawa et al., 2012). Concentrations between 10 and 30 μM IKA were required to reduce uptake of fluorescent nano beads in BY-2 cells and protoplasts (Bandmann and Homann, 2012; Bandmann et al., 2012). In contrast, up to 150 μM IKA had to be used in order to significantly reduce the internalization of styryl dyes (presumably constitutive endocytosis) in *Chara* internodes. This is partly due to the length of treatment, because short term exposure to drugs allows the use of higher concentrations than long-time exposure (compare to Sommer et al., 2015). It could, however, also indicate that different kinds of clathrin components with different susceptibility to IKA, are involved in charasome degradation and constitutive endocytosis.

### Specific membrane sterol composition required for clathrin-dependent charasome dismantling and plasma membrane recycling

A balanced membrane sterol composition was shown to play an essential role in the acquisition of PIN polarity in *A. thaliana* roots (Men et al., 2008) and in auxin signaling-dependent regulation of PIN2 endocytosis (Pan et al., 2009). In plants, sterols occur as a complex mixture of mainly 24-ethyl sterols (sitosterol and stigmasterol) and 24-methyl sterols, cholesterol being only a minor component. Considering that the sterol composition of *C. australis* is similar to higher plants (Patterson et al., 1991), it seemed likely that sterols might play a role in the endocytic events involved in charasome degradation.

Our electron microscopy images and the results of our IKA experiments suggest that clathrin-dependent endocytosis is the main pathway for charasome degradation. However, pharmacological interference using sterol complexing agents also significantly inhibited charasome degradation. This finding indicates the involvement of a sterol-dependent and possibly clathrin-independent endocytic pathway (e.g., Grebe et al., 2003; Boutté et al., 2011; Bandmann et al., 2012). Although we cannot fully exclude this possibility (see below), our electron micrographs of untreated and filipin-treated cells suggest that a specific membrane sterol composition is required for the successful attachment of clathrin (compare to Rodal et al., 1999; Boutté et al., 2010). Further support for this hypothesis comes from the results of our inhibitor treatments. IKA and sterol-complexing agents both reduced the extent of charasome degradation during dark incubation, but when applied simultaneously, no additive effect was observed.

Similar results, i.e., the absence of an additive effect of IKA and sterol complexing agents, were obtained when constitutive endocytosis was analyzed via uptake of styryl dyes. A recent study shows that uptake of FM4-64 in root epidermal cells occurs via clathrin-dependent and clathrin-independent endocytosis (Baral et al., 2015) because of an additive effect of 1-naphthalene acetic acid (NAA), which diminishes clathrin-dependent endocytosis, and MßCD, which complexes the sterols. We found no such additive effect in internodal cells treated with IKA and MßCD, indicative of clathrin representing the major endocytic pathway. This finding suggests that in *Chara*, a specific sterol content of the smooth plasma membrane might be required for successful clathrin coat formation and release of CVs. In favor of this hypothesis, we mention that Ferguson and co-workers found reduced dynamics of clathrin coated structures upon cholesterol depletion in *Drosophila* embryos (Ferguson et al., 2016). A similar stabilization of clathrin-coats was observed in IKA-treated pollen tubes (Moscatelli et al., 2007, see above).

Remarkably, however, neither constitutive endocytosis nor charasome degradation was fully inhibited by IKA and/or sterol complexing dyes, and this indicates that either a residual pathway is still active, or that a minimum residual membrane recycling is necessary for the survival of cells. It is also possible, that the inhibition of clathrin-dependent endocytosis promotes clathrin-independent endocytosis or vice versa (Dutta et al., 2012). The identification and contributions of clathrin-independent endocytic routes, and the interaction between various pathways for early stages in membrane recycling, will be a major challenge for further research (compare Men et al., 2008).

### The clathrin proteins of *Chara australis*

The coat of CPs and vesicles consists of two major components: clathrin and adaptor protein (AP) complexes. In this study we focused on the large clathrin complex, which is composed of heavy and light chains. CHCs comprise an N-terminal region containing clathrin propeller repeats (pfam01394), which extend outwards and are used as a region for binding to other proteins (e.g., ter Haar et al., 1998; Lemmon and Traub, 2012). This binding region is followed by a heavy chain linker (pfam09268), an H-link (pfam13838), and seven clathrin regions (pfam00637), each about 140 amino acids long which form the so called “arm region” (compare Figure 7B). As expected, two CHCs were detected in *C. australis*. They show many similarities to CHCs of other plants, like amino acid composition, molecular mass, and domain arrangement. Nonetheless, CaCHC2 shows less sequence homology to CHC1 and CHC2 proteins of e.g., *A. thaliana, S. moellendorfii*, and *P. patens*, pointing to a possible variation in function. Furthermore, CaCHC2 shows a bindin domain like stretch (pfam02084) of about 100 amino acids at the C-terminus, which is known to form the central hub of the triskelion. In their mature form, bindin proteins comprise almost entirely of this domain, and are involved in sperm to egg fusion. They are known to act as membrane fusogens, as they increase vesicle aggregation by associating specifically with phospholipid vesicles *in vitro* (e.g., Glabe and Vacquier, 1977; Vacquier and Moy, 1977; Glabe, 1985; Zigler, 2008). This interaction is achieved by binding to sulfated fucans, as well as directly to the phospholipid bilayer (Miraglia and Glabe, 1993). To our knowledge, sulfated fucans have not been described in *C. australis* and therefore, the role of the bindin-like domain in this alga remains to be explored.

CaCHCs were classified within the family of plant and fungi CHCs via phylogenetic analyses. As shown in Figure 8, fungi CHC sequences form an outgroup, while the plant sequences fall into several major groups: green algae, Charophyta, ferns and mosses, and higher plants. Clade C contains CaCHC1 and CaCHC2 and shows a closer relationship to land plants than to other green algae. Interestingly, *K. flaccidum* did not cluster with the other Charophyta sequences of clade C, but shows sister relations to *C. australis* and other land plants. But it has to be stated, that the relationship of *K. flaccidum* remains uncertain in this phylogenetic analysis because of low branch support (red circle in Figure 8), which means that this specific clustering of *K. flaccidum* is statistically not fully supported. Moreover, the *Selaginella* sequence XP_002981797 did not group together with other fern and moss sequences. This can be due to different amino acid compositions, as well as a slightly shorter sequence (1695 amino acids compared to 1700 of the other *Selaginella* CHCs). While the two other *Selaginella* proteins demonstrated 99% sequence homology, XP_002981797 showed only 71% to both of them.

The CLCs of *C. australis* (CaCLC1a/b/c and CaCLC2a/b) are much more divergent than the heavy chains, and show a range of 26–93% amino acid identity. For comparison, *A. thaliana* CLCs show 37–49% identities. Moreover, the three described light chains of *A. thaliana* are encoded by three different genes (AtCLC1: At2g20760, accession number Q9SKU1; AtCLC2: At2g40060, accession number O04209; AtCLC3: At3g51890, accession number F4J5M9), and to our knowledge, no alternative splicing was described so far. However, it seems that CaCLCs show variants as CaCLC1a, b, and c, as well as CaCLC2a and b (compare Figures 9A,B). At present, we are not able to link these sequences to any genes, as there are no genomic data available, but we suggest at least two genes and a possible involvement of alternative splicing. It is well known, that mammalian CLCs are alternatively spliced, especially those in brain tissue (e.g., Stamm et al., 1992; Daoud et al., 1999). We searched the available databases for alternative forms of CLC proteins in several plant species. Neither *A. thaliana*, nor *P. patens* revealed clear hints for CLC variations, but we found several highly similar protein sequences in *S. moellendorfii*, that could be alternatively spliced. The presence of different splice variants would strongly suggest functional and/or cell specific differences.

During the course of this study, we were able to demonstrate the crucial role of clathrin in the dark-induced degradation of charasomes, the convoluted plasma membrane areas in *Chara* internodal cells. Molecular analysis of clathrin showed differences in the amino acid composition of one of the two CHCs as well as CLC variants, which may be produced by alternative splicing. In spite of this progress, many questions remain open. For instance, what are the signals responsible for charasome degradation? What is the significance of the clathrin protein variants? Are different variants involved in charasome degradation and constitutive CMEs? In view of these interesting issues, we are convinced that characean internodal cells will remain a valuable model for studying vesicular trafficking.

## Author contributions

IF planned the study. MCH planned the molecular part of the experiments. IF, AS, and MH performed all experiments including inhibitor treatment, microscopic, and statistical analyses. MCH carried out all cloning, sequencing, and protein analysis experiments. CH performed the phylogenetic analyses. IF supervised the project. IF and MCH wrote the manuscript. All authors read and approved the final manuscript.

## Funding

This research was funded by the Austrian Science Fund (FWF; project no. P 27536 to IF).

### Conflict of interest statement

The authors declare that the research was conducted in the absence of any commercial or financial relationships that could be construed as a potential conflict of interest.

## Supplementary material

Supplementary Table 1 **Primer list**. List of primers used for cloning of clathrin heavy chain 1 and 2 as well as all clathrin light chains of *Chara australis* described in this study.

Supplementary Figure 1 **Effect of dark incubation on relative charasome number (A)** and on charasome size **(B)** in branchlet internodal cells of *C. australis*. Cells were incubated in darkness for 12 days, controls were exposed to standard light/dark conditions for 12 days. The box-and-whisker plots show the median values with upper and lower quartiles (boxes), whiskers indicating the 10^th^ and 90^th^ percentiles, and outliers (dots) with *n* = 18 (for dark incubated cells) and 19 (for cells exposed to standard light/dark conditions). Differences between median values of control and treated cells are significant (asterisks; Mann-Whitney Rank Sum Test, *P* ≤ 0.001).

Supplementary Figure 2 **Effect of filipin on the fine structure of Chara internodal cells. (A)** Cell treated with 1.5 μM filipin for 30 min before fixation. **(B)** Untreated cell fixed in the presence of 0.75 μM filipin. Stars indicate charasomes, black arrows point to the plasma membrane. Ch, chloroplast; m, mitochondrion. Bars are 500 nm.

Supplementary Figure 3 **Colocalization of charasomes and clathrin epitopes was quantified using the JACoP plugin of ImageJ (French et al., 2008)**. The scatterplots show the fluorescence intensities (0–254) for each pixel (y-axis = green anti-clathrin fluorescence, x-axis = red charasome fluorescence). Note the absence of colocalization in cells exposed to standard light/dark conditions **(A)** and partial colocalization after 3 days dark incubation **(B)**. Data are based on measurements from 380 **(A)** and 257 **(B)** fluorescent structures, respectively.

Supplementary Video 1 **FM1-43-stained organelles in cell which was treated with 1 μM IKA during 12 dark incubation**. Images were taken at 2 s interval; play back is seven frames per second.

Supplementary Video 2 **FM1-43-stained organelles in cell which was treated with 150 μM IKA for 30 min**. Images were taken at 0.14 s interval; play back is seven frames per second.

## Abbreviations

MW: molecular weight
CP: (clathrin) coated pit
CV: (clathrin) coated vesicle
DRP: dynamin related proteins
TGN: trans Golgi network
CHC: clathrin heavy chain
IKA: ikarugamycin
MßCD: methyl-ß-cyclodextrin
CLC: clathrin light chain
CME: clathrin mediated endocytosis.
