# Supplementary material for: Clathrin in Chara australis: Molecular Analysis and Involvement in Charasome Degradation and Constitutive Endocytosis
Source: Front Plant Sci. 2017 Jan 26;8:20. doi: 10.3389/fpls.2017.00020 (PMC5266738; doi:10.3389/fpls.2017.00020)
Supplement: Supplementary Table 1 — Primer list. List of primers used for cloning of clathrin heavy chain 1 and 2 as well as all clathrin light chains of Chara australis described in this study. [file Table1.PDF]

Supplementary Material

Clathrin in *Chara australis*: Molecular Analysis and Involvement in Charasome Degradation and Constitutive Endocytosis

Marion C. Hoepflinger*, Margit Hoeftberger, Aniela Sommer, Christina Hametner, Ilse Foissner*

*** Correspondence:** Marion C. Hoepflinger: Marion.Hoepflinger2@sbg.ac.at
Ilse Foissner: Ilse.Foissner@sbg.ac.at

1 Supplementary Table

Primer list - *Chara australis* clathrin

			name	sequence
**Clathrin Light Chain**	**1a**		CLC_CL671_fwd	5'-ATGGACGATTTTGGAGCGGAGG-3'
			CLC_CL671_rev1	5'-TCAGAAAAAACCCCATTCGGT-3'
	**1b**		CLC_CL671_fwd	*see above*
			CLC_CL671_rev2	5'-TCAGAAAAAGAGATTGTCCCT-3'
	**1c**		CLC_CL671_fwd	*see above*
			CaCLC_rev	5'-TCAGACAAAGACAGACTGCCC-3'
	**2a**		CLC_CL6108_fwd	5'-ATGTCCGAGGAACAGGTGGAG-3'
			CLC_CL6108_rev	5'-CTAAACAACTCCACTATTCTT-3'
	**2b**		CLC_CL6108_fwd	*see above*
			CLC_CU24939_rev	5'-TTACAGTGTCGCAGGTGCTGC-3'
**Clathrin Heavy Chain**	**1**	part 1	CHC_CL2596_fwd	5'-ATGGCGTCCGCTAGTGCTCCTA-3'
			CHC_CL2596_rev2	5'-GCGGGGTCCGACCTCATGATAG-3'
		part 2	CHC_CL2596_fwd2	5'-ACATCAAGGCGAAGGCGACTCC-3'
			CHC_CL2596_rev3	5'-CGAGCCGGTTGACGTAGTCCAT-3'
		part 3	CHC_CL2596_fwd3	5'-TGCCGCACGAGCTCATCGAGCT-3'
			CHC_CL3755_rev	5'-CTAGTAACCTGTCATGGGGGGC-3'
**Clathrin Heavy Chain**	**2**	part 1	CHC_CL32962_fwd	5'-ATGGCGGCTGCCAGCGCCCCCA-3'
			CHC_CL32962_rev2	5'-TGGGAGTACTTCAGAATTCTATC-3'
		part 2	CHC_CL32962_fwd2	5'-GCTTGGAGACCTCGTCAAGCCTG-3'
			CHC_CL32962_rev3	5'-GCAGATTCTGGAGATTCGGATTG-3'
		part 3	CHC_CL32962_fwd3	5'-GCCAGAGAGCAAGAGTCCAGAA-3'
			CHC_U5088_rev	5'-TCAGTACCCAGCCATCTGGGGC-3'

Supplementary Table 1. Primer list. List of primers used for cloning of clathrin heavy chain 1 and 2 as well as all clathrin light chains of *Chara australis* described in this study.
