# Supplementary material for: Clathrin in Chara australis: Molecular Analysis and Involvement in Charasome Degradation and Constitutive Endocytosis
Source: Front Plant Sci. 2017 Jan 26;8:20. doi: 10.3389/fpls.2017.00020 (PMC5266738; doi:10.3389/fpls.2017.00020)
Supplement: Supplementary Figure 1 — Effect of dark incubation on relative charasome number (A) and on charasome size (B) in branchlet internodal cells of C. australis. Cells were incubated in darkness for 12 days, controls were exposed to standard light/dark conditions for 12 days. The box-and-whisker plots show the median values with upper and lower quartiles (boxes), whiskers indicating the 10th and 90th percentiles, and outliers (dots) with n = 18 (for dark incubated cells) and 19 (for cells exposed to standard light/dark conditions). Differences between median values of control and treated cells are significant (asterisks; Mann-Whitney Rank Sum Test, P ≤ 0.001). [file Image1.pdf]

Supplementary Material

Clathrin in *Chara australis*: Molecular Analysis and Involvement in Charasome Degradation and Constitutive Endocytosis

Marion C. Hoepflinger*, Margit Hoeftberger, Aniela Sommer, Christina Hametner, Ilse Foissner*

*** Correspondence:** Marion C. Hoepflinger: Marion.Hoepflinger2@sbg.ac.at
Ilse Foissner: Ilse.Foissner@sbg.ac.at

1.1 Supplementary Figure

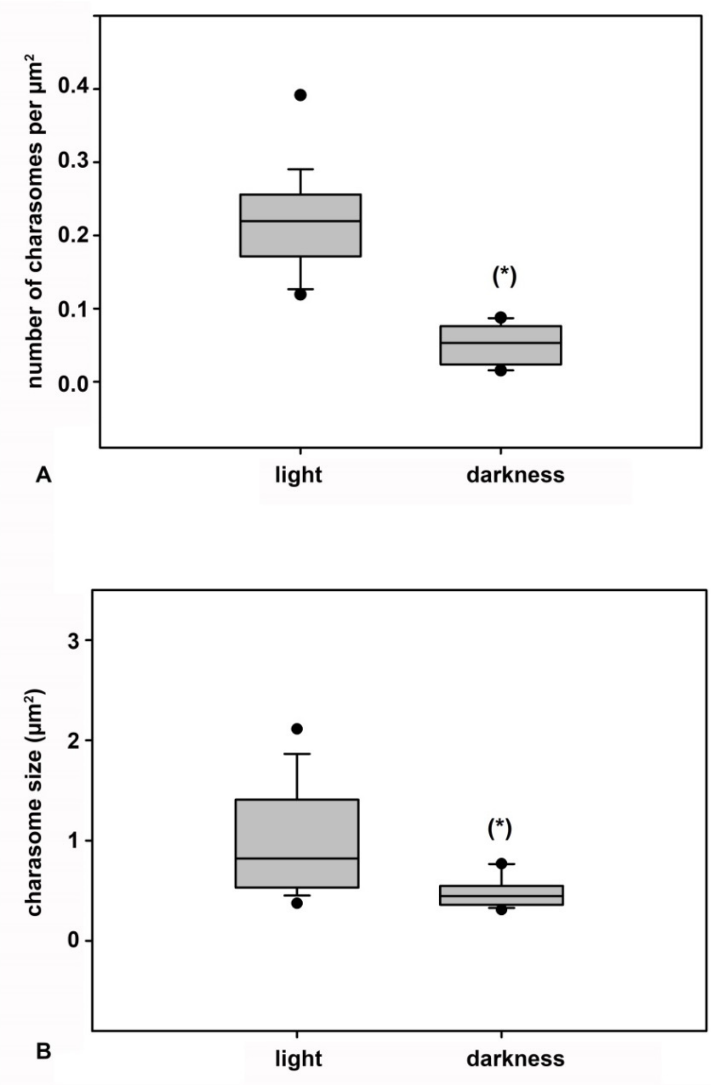

Supplementary Figure 1. Effect of dark incubation on relative charasome number (A) and on charasome size (B) in branchlet internodal cells of *C. australis*. Cells were incubated in darkness for 12 days, controls were exposed to standard light/dark conditions for 12 days. The box-and-whisker plots show the median values with upper and lower quartiles (boxes), whiskers indicating the 10th and 90th percentiles, and outliers (dots) with n = 18 (for dark incubated cells) and 19 (for cells exposed to standard light/dark conditions). Differences between median values of control and treated cells are significant (asterisks; Mann-Whitney Rank Sum Test, $P \leq 0.001$).
