# Supplementary material for: Clathrin in Chara australis: Molecular Analysis and Involvement in Charasome Degradation and Constitutive Endocytosis
Source: Front Plant Sci. 2017 Jan 26;8:20. doi: 10.3389/fpls.2017.00020 (PMC5266738; doi:10.3389/fpls.2017.00020)
Supplement: Supplementary Figure 2 — Effect of filipin on the fine structure of Chara internodal cells. (A) Cell treated with 1.5 μM filipin for 30 min before fixation. (B) Untreated cell fixed in the presence of 0.75 μM filipin. Stars indicate charasomes, black arrows point to the plasma membrane. Ch, chloroplast; m, mitochondrion. Bars are 500 nm. [file Image2.pdf]

Supplementary Material

Clathrin in *Chara australis*: Molecular Analysis and Involvement in Charasome Degradation and Constitutive Endocytosis

Marion C. Hoepflinger*, Margit Hoeftberger, Aniela Sommer, Christina Hametner, Ilse Foissner*

*** Correspondence:** Marion C. Hoepflinger: Marion.Hoepflinger2@sbg.ac.at
Ilse Foissner: Ilse.Foissner@sbg.ac.at

1.1 Supplementary Figure

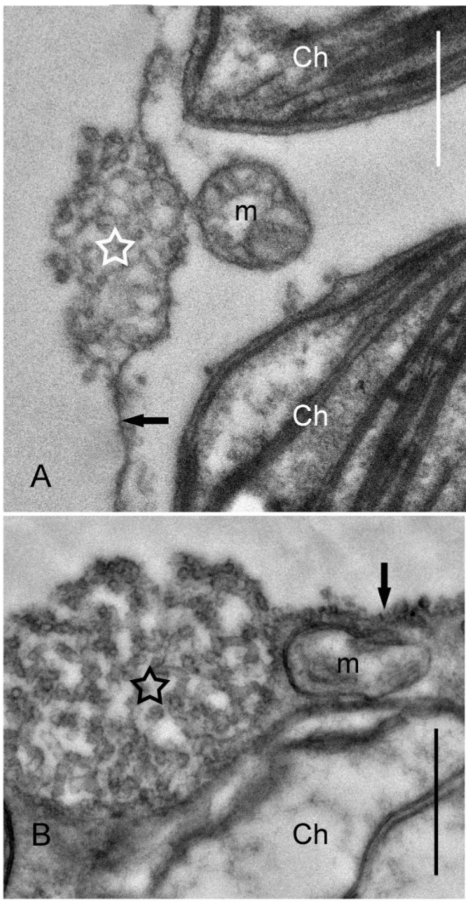

Supplementary Figure 2. Effect of filipin on the fine structure of *Chara* internodal cells. (A) Cell treated with 1.5 µM filipin for 30 min before fixation. (B) Untreated cell fixed in the presence of 0.75 µM filipin. Stars indicate charasomes, black arrows point to the plasma membrane. Ch = chloroplast, m = mitochondrion. Bars are 500 nm.
