# Supplementary material for: Clathrin in Chara australis: Molecular Analysis and Involvement in Charasome Degradation and Constitutive Endocytosis
Source: Front Plant Sci. 2017 Jan 26;8:20. doi: 10.3389/fpls.2017.00020 (PMC5266738; doi:10.3389/fpls.2017.00020)
Supplement: Supplementary Figure 3 — Colocalization of charasomes and clathrin epitopes was quantified using the JACoP plugin of ImageJ (French et al., 2008). The scatterplots show the fluorescence intensities (0–254) for each pixel (y-axis = green anti-clathrin fluorescence, x-axis = red charasome fluorescence). Note the absence of colocalization in cells exposed to standard light/dark conditions (A) and partial colocalization after 3 days dark incubation (B). Data are based on measurements from 380 (A) and 257 (B) fluorescent structures, respectively. [file Image3.pdf]

Supplementary Material

Clathrin in *Chara australis*: Molecular Analysis and Involvement in Charasome Degradation and Constitutive Endocytosis

Marion C. Hoepflinger*, Margit Hoeftberger, Aniela Sommer, Christina Hametner, Ilse Foissner*

*** Correspondence:** Marion C. Hoepflinger: Marion.Hoepflinger2@sbg.ac.at
Ilse Foissner: Ilse.Foissner@sbg.ac.at

1.1 Supplementary Figure

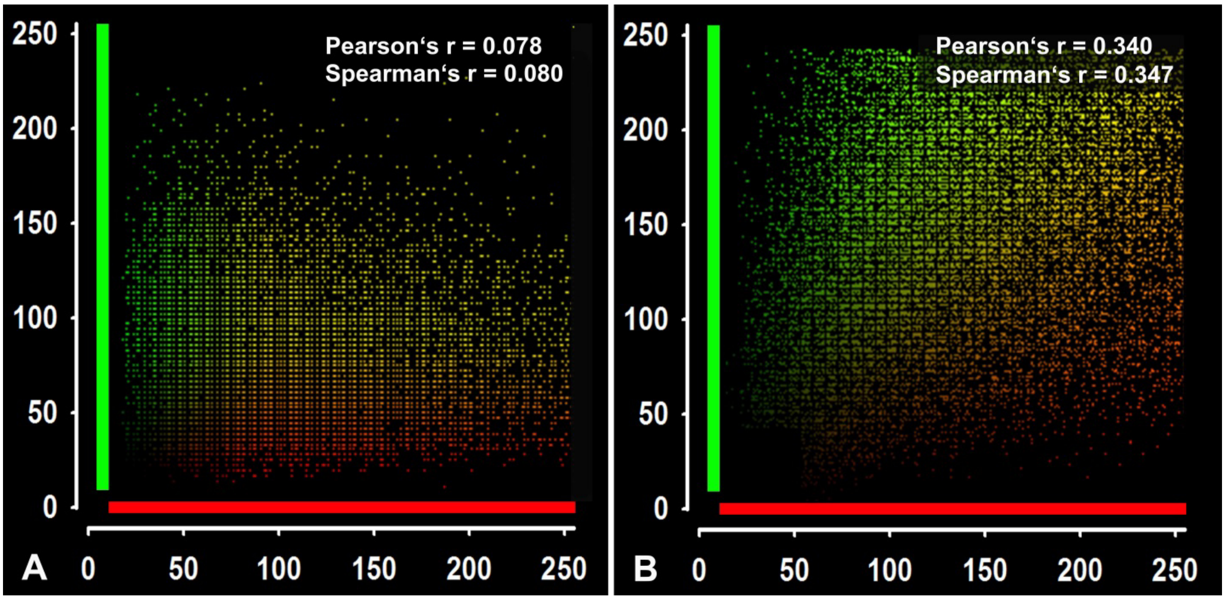

Supplementary Figure 3. Colocalization of charasomes and clathrin epitopes was quantified using the JACoP plugin of ImageJ (French et al., 2008). The scatterplots show the fluorescence intensities (0–254) for each pixel (y-axis = green anti-clathrin fluorescence, x-axis = red charasome fluorescence). Note the absence of colocalization in cells exposed to standard light/dark conditions (A) and partial colocalization after 3 days dark incubation (B). Data are based on measurements from 380 (A) and 257 (B) fluorescent structures, respectively.

Reference:
French, A. P., Mills, S., Swarup, R., Bennett, M. J., and Pridmore, T. P. (2008). Colocalization of fluorescent markers in confocal microscope images of plant cells. *Nat. Protocols* 3(4), 619-628.
